# *Morinda officinalis* Polysaccharides Ameliorates Bone Growth by Attenuating Oxidative Stress and Regulating the Gut Microbiota in Thiram-Induced Tibial Dyschondroplasia Chickens

**DOI:** 10.3390/metabo12100958

**Published:** 2022-10-10

**Authors:** Chaodong Zhang, Tingting Xu, Luxi Lin, Aftab Shaukat, Xishuai Tong, Ke Yue, Qinqin Cao, Cai Zhang, Fang Liu, Shucheng Huang

**Affiliations:** 1 Department of Clinical Veterinary Medicine, College of Veterinary Medicine, Henan Agricultural University, Zhengzhou 450046, China; 2 National Center for International Research on Animal Genetics, Breeding and Reproduction (NCIRAGBR), Huazhong Agricultural University, Wuhan 430070, China; 3 Joint International Research Laboratory of Agriculture and Agri-Product Safety of the Ministry of Education of China, Institutes of Agricultural Science and Technology Development, College of Veterinary Medicine, Yangzhou University, Yangzhou 225009, China; 4 Laboratory of Environment and Livestock Products, Henan University of Science and Technology, Luoyang 471023, China

**Keywords:** gut microbiota, oxidative stress, *Morinda officinalis* polysaccharides, tibial dyschondroplasia, leg disease, Chinese herbal medicine

## Abstract

Tibial dyschondroplasia (TD) occurs in chickens and other fast-growing birds, affecting their cartilage growth and leading to reduced meat quality in broilers. *Morinda officinalis* polysaccharide (MOP) is one of the chief active components of *Morinda officinalis*, which promotes bone formation, inhibiting bone loss and having anti-oxidant and anti-inflammatory properties. A total of 120 AA chickens were randomly divided into the CON group (basal diet), TD group (100 mg/kg thiram + basal diet), and MOP group (100 mg/kg thiram + basal diet + water with 500 mg/kg MOP). The experiment lasted 21 days. The results showed that MOP could alleviates broiler lameness caused by TD, restore the morphological structure of tibial growth plate (TGP), increase tibial weight (*p* < 0.05), balance the disorder of calcium and phosphorus metabolism, and promote bone formation by increasing the expression of BMP-2, Smad4, and Runx2 genes In addition, MOP supplementation stimulated the secretion of plasma antioxidant enzymes (T-SOD and GSH-Px) by regulating the expression of SOD and GPX-1 genes, thereby enhancing the antioxidant capacity of TD broilers. Interestingly, we observed MOP can also improve gut microbiota by increasing the beneficial bacteria count and decreasing the harmful bacteria count. These findings indicated that MOP can regulate bone formation through the BMP/Smads signaling pathway, attenuating oxidative stress and regulating the gut microbiota of TD broilers, so as to achieve the effect of treating TD. This suggests that MOP might be a potential novel drug in the treatment of TD in chickens.

## 1. Introduction

In meat-type poultry, tibial dyschondroplasia (TD) is a common disease that affects leg health. The TD occurs mainly in fast-growing chicken breeds; it is characterized by lesions composed of uncalcified, nonvascularized cartilage in the proximal tibial growth plate (TGP), which leads to lameness due to tibia fracture and bone deformation, thereby reducing performance and compromising animal welfare [1,2,3]. Our recent studies demonstrated that TD broiler chickens have also adverse effects on muscle quality [4,5]. Under the excessive pursuit of economic benefits, with the gradual improvement of intensification of modern broiler breeding, the issue of broiler leg disease has become increasingly prominent. Therefore, TD, as a skeletal disease with a high prevalence in broiler leg disease, proposes a great challenge for its prevention and treatment [6,7]. 

Previous research identified that the signaling pathways of BMP/Smads, Wnt/β-catenin, and OPG/RANKL/RANK play a chief role in bone metabolism [8,9]. BMP/Smads signaling pathway is the key signaling pathway in bone formation and has been confirmed to affect osteoblast differentiation [10]. The Smad protein, an intracellular signaling protein, is the key intermediate of the canonical transforming growth factor-beta (TGF-β) signaling pathway and BMP pathway, regulating the growth, development, regeneration, and repair of bone [11]. BMP-2 binds to cell-surface receptors during bone formation, which is then picked up by other Smad molecular receptors in the Smad family and activated. If activated, then BMP-2 will bind to the Smad family member 4 (Smad4) to form a complex for cytoplasmic transfer [12,13,14]. In addition, the runt-related transcription factor-2 (Runx2) also plays a critical role in bone formation regulated by osteoblast differentiation, which depends on the Smad interaction with BMP-2 and TGF-β [15]. Therefore, understanding the potential role of BMP/Smads signaling in bone formation may contribute to implicate the pathogenesis of TD in chicken. Additionally, oxidative stress and gut microbiota are involved in the development of a variety of diseases, including bone diseases [16,17,18,19,20,21]. Excessive reactive oxygen species (ROS) attenuate bone marrow mesenchymal stem cell (BMSC) directed differentiation into osteoblasts and inhibit their proliferation through the ROS/FoxO/PPARγ/β-catenin pathway, and induce osteoblast apoptosis through the PKCβ/p53/P66SHC/JNK pathway. By activating the JNK pathway and inhibiting the Akt pathway, TCF/LEF transcription in the Wnt/β-catenin signaling pathway can be transferred to FoxO transcription, which can reduce bone formation of osteoblasts. Moreover, excessive ROS up-regulate the expression of RANKL on the surface of bone formation-related cells, promote the differentiation and maturation of osteoclasts, and directly or indirectly participate in the degradation of bone matrix, finally breaking the balance of bone reconstruction between bone resorption and bone formation. [22,23,24,25,26]. Importantly, the gut microbiota (GM), made up of trillions of bacteria or molecules they synthesize in the gastrointestinal tract, plays an important role in bone health [27,28]. Numerous studies have depicted that the effects of gut microbiota on bone metabolism involve three potential mechanisms: regulating the absorption of nutrients by intestinal epithelial cells; regulating the mucosal and systemic immune system; transport of gut microbiota metabolites across intestinal endothelial barrier [29,30,31]. The dysfunction or disorder of gut microbiota may cause bone diseases such as osteoporosis (OP) and osteoarthritis (OA) or skeletal dysplasia through these potential mechanisms [19,21,32,33]. However, the oxidative stress and the gut microbiota that interact with the TD broilers remain unknown.

Traditional Chinese medicine (TCM) has been widely used to prevent and treat diseases due to its efficacy with less toxicity and fewer side effects [34]. *Morinada officinalis* polysaccharide (MOP) is a derivate extracted from *Morinada officinalis*, which is widely distributed in nature and has anti-oxidative, anti-inflammatory properties, and has also been reported to promote bone formation [35]. Our previous study proved that MOP can improve TD broiler meat quality by reducing oxidative damage [5], but its therapeutic pathway for TD remains to be studied. Zhang et al. [34] reported that MOP could promote bone formation by regulating the BMP-2/Runx-2/Osterix pathway. The skeletal development stage of broilers is 0–3 weeks of age, and from 3 weeks of age to market stage, they are in a rapid weight gain stage. This study aimed to investigate the effects and the potential mechanism of MOP on the tibial growth, oxidative stress, and gut microbiota homeostasis of TD broiler chickens during the skeletal development stage, which may provide new insight for the application of MOP in chicken leg diseases.

## 2. Materials and Methods

### 2.1. Animal Ethics

We followed the animal welfare guidelines of the College of Animal Science and Veterinary Medicine of Henan Agricultural University Zhengzhou, China in all of our broiler experiments. (License No. 170126).

### 2.2. Chemicals, Animals, and Experimental Protocol

Tetramethylthiuram disulfide (Thiram, #M03718066) was purchased from Macklin Biochemical Co., Ltd. (Shanghai, China). MOP (#CY180812) was acquired from Yangling Ciyuan Biotechnology Co., Ltd. (Shanxi, China). A total of 120 Arbor Acres chickens (1-day-old; 46.96 ± 6.53 g) were obtained from XingDa Poultry Industry Co., Ltd. (Kaifeng, China).

**Figure 1:**
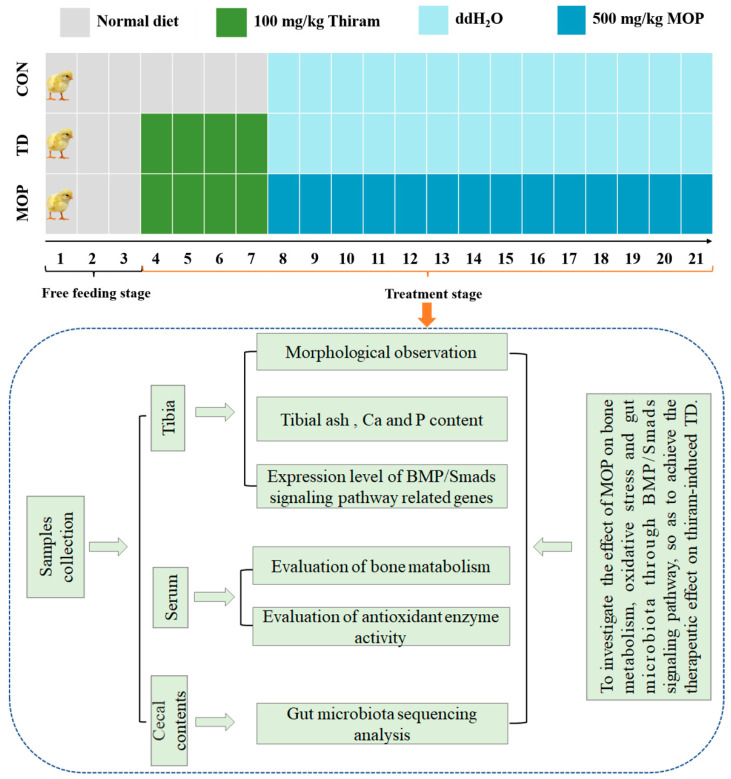
Experimental design diagram of MOP for treatment of thiram-induced tibial dyschondroplasia in broilers. ddH2O: double distilled water; MOP: *Morinda officinalis* polysaccharides.

All broilers were housed in single-layer metal cages with standard room temperature, relative humidity, and well-ventilated light/dark cycles. The room temperature was controlled from 33 °C to 35 °C during the first week and gradually decreased to 28 °C for the rest of the experiment. During the whole experimental phase, the ambient humidity was controlled between 60–70%, and the daily illumination time was fixed as 23 h illumination time and 1 h light-off time. Broilers were fed and watered freely. Broilers were randomly divided into 3 groups with 4 replicate cages in each group and 10 chickens in each replicate cage, which were control group (CON), Thiram-induced TD group (TD), and MOP treatment group (MOP). Broilers in the CON group were fed a normal diet (feed nutrient composition analysis is shown in Table 1), and broilers in experimental groups (TD and MOP) were fed the same diet as those in the CON group and were supplemented with 100 mg/kg thiram from days 4 to 7. On days 7–21, 500 mg/kg MOP was added to the drinking water in the MOP group (water was cut off for 1 h at a fixed time every day, and then drinking water mixed evenly with MOP was provided until it was finished, then normal drinking water replaced it). All experiments lasted for 21 days, and the body weight of broilers was measured every day (Figure 1). Corn-soybean meal-based diets were formulated to meet or exceed the feeding standard of China for broiler chickens (NY/T33-2004, Ministry of Agriculture of the People’s Republic of China, 2004). The diet did not contain any supplemented antibiotics. The composition of the diet and nutrient levels are presented in Table 1.

### 2.3. Determination of Tibia Indicators

On days 7, 14, and 21, the prevalence of TD in broilers was calculated by visually observing whether the broilers developed swollen joints and difficult walking or standing behavior. A total of ten chickens from each group were sacrificed on days 7, 14, and 21 through cervical dislocation, and then their entire tibias were dissected and weighed by electronic balance. The left proximal tibia was sectioned lengthwise to expose the tibial growth plate, the tibial length and growth plate width were accurately measured using vernier calipers. Tibia weight index and growth plate width coefficient were calculated as follows: Tibial weight index = tibial weight/broiler body weight; tibial growth plate index = tibial growth plate width/tibial length.

### 2.4. Determination of Tibia Ash and Calcium and Phosphorus Content

The right tibia was taken from 6 chickens in each group. The excess tissue on the surface of the tibia was removed, the bone marrow cavity was cleaned, and the tibia was placed in a hot blast furnace. The tibia was dried to constant weight and then crushed. The tibia was placed in a ceramic crucible, roasted at 550–600 °C for 3 h, and cooled to a constant weight, namely the ash weight of the tibia. The contents of calcium (Ca) and phosphorus (P) in the tibia were determined by EDTA complexometric titration and the vanadium molybdenum yellow colorimetric method. Tibial ash content = tibial ash weight/tibial weight after drying.

### 2.5. Determination of Plasma Biochemical Indicators

The plasma samples were taken via centrifugation at 3000 rpm for 10 min at 4 °C and stored at −20 °C after blood samples were taken aseptically from the carotid vein of ten chicks at 7, 14, and 21 days for each group. The plasma for the content of Ca, P, alkaline phosphatase (ALP) and the activities of total superoxide dismutase (T-SOD) and glutathione peroxidase (GSH-Px), and the concentration of malondialdehyde (MDA) were determined using a commercial ELISA kit according to the manufacturer’s instructions (Nanjing Jiancheng Bioengineering Institute, Nanjing, China). 

### 2.6. Histopathological Analysis

Left tibia samples of broilers were fixed in 4% paraformaldehyde solution on days 7, 14, and 21, and were completely decalcified with 10% EDTA decalcification solution. The decalcified tibial sample was dehydrated in ethanol and cleaned with xylene, then embedded in paraffin, and made into 5 μm thick tissue sections. Finally, hematoxylin and eosin (H&E) staining was performed as described by Huang et al. [36].

### 2.7. RNA Isolation and RT-qPCR

Total RNA of the right tibial growth plate was isolated using trizol reagent (Dalian Takara Biotechnology Co., Ltd., Dalian, China). The primers of BMP/Smads signaling-related genes (BMP-2, Smad4, Runx2), oxidation-related genes (SOD, GPX-1), and GAPDH mRNA sequences were designed and synthesized by Primerbank, TSINGKE Biological Technology Co., Ltd. (Zhengzhou, China). All the primer sequences employed in this study are shown in Table S1. RT-qPCR was performed according to SYBR Green I PCR Master Mix (Vazyme Biotech Co., Ltd., Nanjing, China). The reaction conditions are as follows: pre-denaturation at 95 °C for 30 s, deformation at 95 °C for 10s, extension at 72 °C for 30 s, annealing at 60 °C for 30 s, a total of 40 cycles. Relative gene expression levels were analyzed by the 2^−ΔΔCt^ method after normalization against GAPDH.

### 2.8. Gut Microbiota Sequencing Analysis

On day 21, four cecal contents of broilers in each group were collected and stored at −80 °C, and then were separately thawed on ice, homogenized, and microbial genomic DNA was extracted from samples using the QIAamp DNA tool mini-kit (Qiagen, Hilden, Germany), following the manufacturer’s protocol. To ensure successful DNA isolation, Nanodrop instruments (Thermo Fisher Scientific, Waltham, MA, USA) were used to measure the concentration and purity of the DNA extract to determine whether the DNA was successfully isolated and to evaluate the integrity of the DNA sample by 1% agarose gel electrophoresis. The V3-V4 hypervariable region of bacterial 16S rRNA was amplified by PCR with forward primer 338F (5′-ACTCCTACGGGAGGCAGCA-3′) and reverse primer 806R (5′-GGACTACHVGGGTWTCTAAT-3′). Amplifiers were purified and quantified using Agcourt AMPure Beads and PicoGreen dsDNA detection kits (Invitrogen, Carlsbad, CA, USA). Then, DNA libraries were constructed, and PCR amplification products were sequenced on Illumina Miseq 250 platform (Illumina Inc., San Diego, CA, USA). Alpha diversity was assessed by Chao1, Fisher and Shannon index analysis. β-diversity was assessed by principal coordinate analysis (PCoA) to analyze the spatial distribution and distance between samples. In addition, linear discriminant analysis effect Size (LEfSe) analysis and other data from 16s sequencing were analyzed by genetic cloud tools (a free online data analysis platform, http://www.genescloud.cn, accessed on 1 December 2021).

### 2.9. Statistical Analysis

All experimental data were analyzed by SPSS 26.0 software (SPSS Incorporated, Chicago, IL, USA) or Graphpad Prism software (#Version 8.00, GraphPad Software, Inc. La Jolla, CA, USA). Oxidative stress, bone-related indices, and microbiota richness were statistically analyzed by unpaired two-tailed Student’s t-test and one-way ANOVA with LSD post-hoc test. Principal analysis (PCA) of related genes was performed to assess pairwise distances among samples using cloud tools (a free online platform for data analysis, https://www.omicstudio.cn/tool, accessed on 1 November 2021). Inter-data Pearson and Spearman correlation analysis was performed using a psych package in R (https://www.r-project.org, accessed on 1 December 2021). The experimental data were presented as mean ± SD. *p* < 0.05 was considered statistically significant. 

## 3. Results

### 3.1. Effect of MOP on Clinical Symptoms, Tibial Parameters, Histopathology of TGPs in TD Broilers

**Figure 2:**
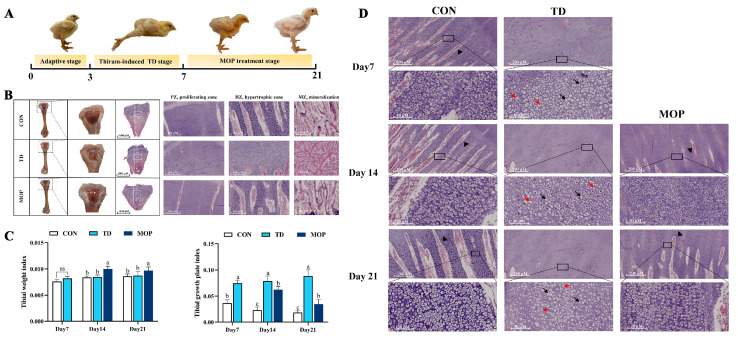
(**A**) Clinical characteristics of broiler chickens in adaptation stage, thiram-induced TD stage and MOP treatment stage. (**B**) Morphological alteration of tibia and tibial growth plate, HE staining of the tibial growth plate. BV, blood vessel; GP, growth plate; TDL, tibial dyschondroplasia lesion. AC, articular cartilage; HZ, hypertrophic zone; PZ, proliferation zone. MZ, mineralization zone. (**C**) Tibial growth plate index and tibial weight index. Bars with no common small letters (a, b, c) indicate statistical differences among the three treatments (*p* < 0.05). ns showed no statistical significance among the three treatment groups (*p* > 0.05). (**D**) The histopathological examination of vascular distribution and cell morphology in hypertrophic zone of tibial growth plate on various days 7, 14, and 21. Black arrow, nuclear lysis; Red arrow, nuclear fragmentation; Black triangle, blood vessel.

It was observed that no broilers in the CON group developed TD on days 7, 14, and 21. On day 7, the prevalence of TD was 100% in both TD and MOP groups, 90% and 27.7% in TD and MOP groups on day 14, and 80% and 15% in TD and MOP groups on day 21. The clinical symptoms of broiler chickens were reduced or had lost appetite, with swollen tibial joints, difficulty in standing and walking, or even inability to stand and walk after establishing the TD broiler model. After MOP treatment, the appetite began to recover, joint swelling was reduced, and the ability to stand and walk freely was gradually restored for TD lesions in broilers (Figure 2A). The tibial growth plate of broilers in the CON group had normal morphology, with a large number of vascular infiltrated in the hypertrophy zone (HZ), and the trabecular bone in the proximal tibia in the mineralization zone (MZ) was thick and closely arranged. The trabeculae joined into a network. Compared with the CON group, the TGP of the TD group was significantly thickened, and transparent white “cartilage thrombus” was seen. There was almost no vascular infiltration in the hypertrophy zone, and the thickness of the proximal tibial trabecular bone in the mineralization zone was significantly reduced and sparsely arranged. After MOP treatment, TGP of TD broilers gradually returned to normal, blood vessels increased significantly in the hypertrophic zone of proximal tibia, and trabecular bone thickness increased significantly in the mineralized zone, and the arrangement was dense (Figure 2B). Next, the tibial parameters, including tibial weight and tibial growth plate width were measured as revealed in Figure 2C. The results exhibited no significant difference in tibial weight index between TD and CON groups. After MOP treatment, the tibial weight index of broilers in the MOP group was significantly higher than that in the TD group on day 14 (*p* < 0.001) and day 21 (*p* = 0.014). The TGP index of the TD group was significantly higher than that of the CON group due to the presence of a white “cartilage plug” on days 7, 14, and 21 (*p* < 0.001, *p* < 0.001, and *p* < 0.001, respectively). After MOP treatment, the width coefficient of the TGP in the MOP group showed a positive trend of decline. The results indicated that MOP could improve tibia development by affecting tibia weight and growth plate width.

Further histopathological analysis of HZ of tibial growth plate showed a significant decrease in the number of blood vessels and chondrocytes, with a disorganized distribution of chondrocytes and incomplete cell morphology with nuclear lysis and nuclear fragmentation in the TGP of the TD group compared to the CON group. In the MOP-treated group, there was more vascular infiltration in the tibial growth plate than in the TD group. In addition, the chondrocytes in the MOP group gradually repaired and regenerated in a neat and orderly arrangement and with only a few empty nucleated cells (Figure 2D). These results suggested that MOP can restore TD injury by improving TGP structure and enhancing vascular infiltration of the TGP in broiler chickens.

### 3.2. Effect of MOP on Calcium, Phosphorus, and Ash Content in Tibia and Plasma of TD Broilers

**Figure 3:**
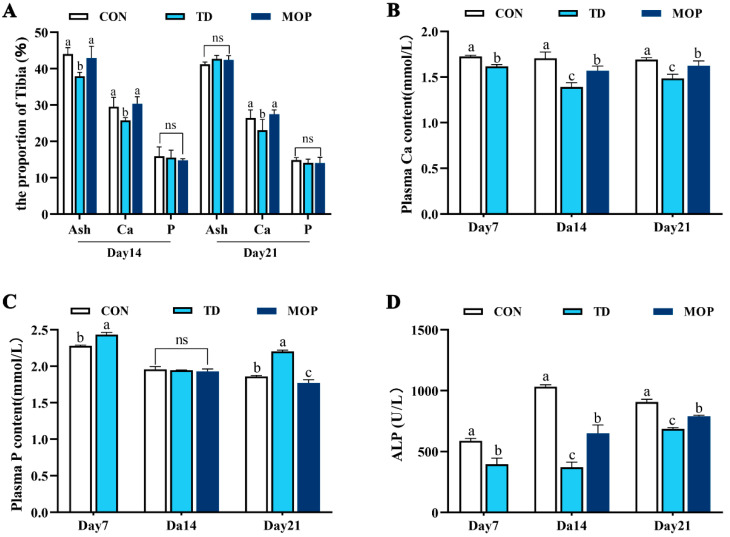
Effects of MOP on tibial ash, calcium (Ca) and phosphorus (P) levels and plasma bone metabolism indices of TD broilers. (**A**) The contents Ca, P, and ash in the tibia on days 14 and 21. (**B**) Plasma Ca content. (**C**) Plasma P content. (**D**) Plasma alkaline phosphatase (ALP) levels. Bars with no common small letters (a, b, c) indicate statistical differences among the three treatments (*p* < 0.05). ns showed no statistical significance among the three treatment groups (*p* > 0.05).

Tibial ash content and inorganic mineral content are closely related to bone. To investigate the effect of MOP on the ash content and bone calcium and phosphorus content of TD broiler tibiae, tibiae samples from day 14 and day 21 were measured (Figure 3A). The results revealed that the tibial ash content was significantly lower in the TD group than in the CON and MOP groups on day 14 (*p* = 0.006 and *p* = 0.016, respectively), and the difference was not significant on day 21. Similarly, the tibial Ca content in the TD group was significantly lower than that in the CON and MOP groups on day 14 (*p* = 0.042 and *p* = 0.030, respectively) and day 21 (*p* = 0.034 and *p* = 0.010, respectively). Additionally, there was no significant difference in tibial P content between the groups. These results indicated that MOP could improve bone mass by increasing ash content and Ca content of the tibia.

In addition, biochemical indices of plasma ALP, Ca, and P were further examined in each group (Figure 3B–D). Compared with the CON group, plasma Ca and ALP levels in the TD group were significantly decreased on day 7 (*p* = 0.001 and *p* = 0.003, respectively), day 14 (*p* < 0.001 and *p* < 0.001, respectively), and day 21 (*p* = 0.003 and *p* < 0.001, respectively). However, plasma P levels were significantly higher in the TD group than in the CON group on days 7 and 21 (*p* = 0.008 and *p* < 0.001, respectively). After MOP treatment, plasma Ca and ALP levels in the MOP group were significantly higher than those in the TD group on day 14 (*p* = 0.008 and *p* = 0.046, respectively) and day 21 (*p* = 0.046 and *p* < 0.001, respectively). On the contrary, plasma P levels in the MOP group were significantly lower than in the TD group on day 21 (*p* < 0.001). The results demonstrated that MOP could alleviate the disturbance of Ca and P caused by TD, increase ALP activity, a marker of bone formation, and thereby promote bone formation.

### 3.3. Effect of MOP on the Expression of BMP/Smads Signaling Pathway in TD Broilers

**Figure 4:**
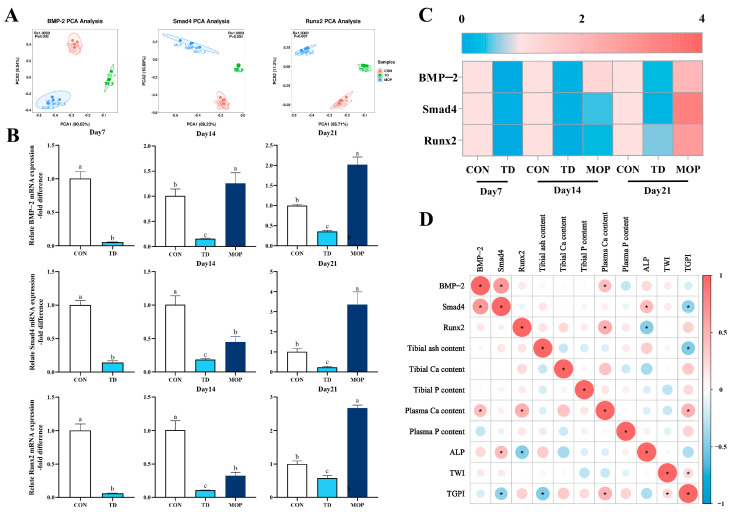
Effects of MOP on bone formation-related genes in TD broilers. (**A**) PCA of the BMP-2, Smad4, and Runx2 gene expressions with an unsupervised pattern recognition method. (**B**) BMP-2, Smad4, and Runx2 gene expression. (**C**) Heatmap shows the expression of genes associated with antioxidant activity. (**D**) Pearson correlation analysis between BMP/Smads signaling pathway and bone metabolism-related parameters. TWI: Tibial weight index; TGPI: Tibial plate index. Bars with no common small letters (a, b, c) indicate statistical differences among the three treatments (*p* < 0.05).

To investigate the effect of MOP on the expression of BMP-2, Smad4, and Runx2 genes on the TGP of broiler chickens, PCA was performed using an unsupervised pattern recognition to assess the clustering of gene expression in the three groups and the results showed that the PCA score plot of BMP-2, Smad4, and Runx2 genes were highly dispersed as in Figure 4A. Further analysis of the expression levels of BMP-2, Smad4, and Runx2 genes in the TGP revealed that the expression of BMP-2, Smad4, and Runx2 genes markedly decreased in the TD group compared to the CON group on day 7 (*p* < 0.001, *p* < 0.001 and *p* < 0.001, respectively), day 14 (*p* < 0.001, *p* < 0.001, and *p* < 0.001, respectively) and day 21 (*p* < 0.001, *p* = 0.047, and *p* < 0.001, respectively). After MOP treatment, the expression levels of BMP-2 Smad4 and Runx2 genes were significantly higher in the MOP group than in the TD group on day 14 (*p* < 0.001, *p* = 0.011, and *p* < 0.001, respectively) and day 21 (*p* < 0.001, *p* < 0.001, and *p* < 0.001, respectively) (Figure 4B). In addition, the heatmap showed more clearly the differences in gene expression levels in each group (Figure 4C). Pearson correlation analysis explored the correlation between BMP/Smads signaling pathway and bone metabolism-related parameters (Figure 4D). The results found that plasma Ca level was significantly positively correlated with BMP-2 (r = 0.421, *p* = 0.040) and Runx2 (r = 0.537 and *p* = 0.007); TGPI (tibial growth plate index), an indicator of tibial growth plate injury, was negatively correlated with Smad4 gene (r = −0.421, *p* = 0.040); plasma ALP actively was significantly correlated with Smad4 genes (r = 0.440, *p* = 0.031), and negatively correlated with Runx2 gene (r = −0.478, *p* = 0.031). These results suggested that MOP may regulate the calcium homeostasis through the BMP/Smads signaling pathway, thereby improving the structure of the TGP in TD broiler chickens.

### 3.4. Effect of MOP on Antioxidant Activity in TD Broilers

**Figure 5:**
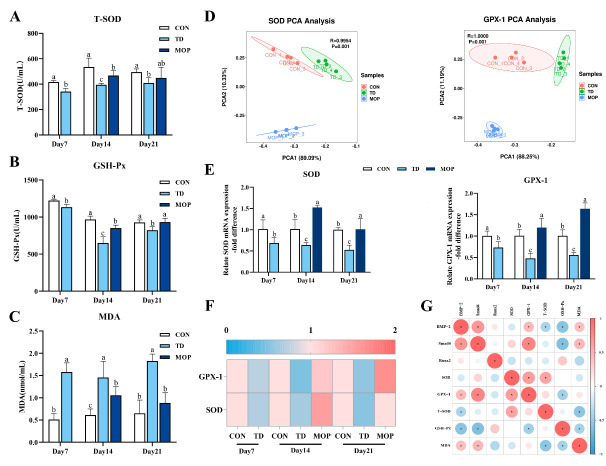
Effect of MOP on the antioxidant ability of broilers. (**A**) Plasma T-SOD activity. (**B**) Plasma GSH-Px activity. (**C**) Plasma MDA level. (**D**) PCA of the SOD, GPX-1 gene expressions with an unsupervised pattern recognition method. (**E**) SOD, GPX-1 gene expression. (**F**) Heatmap shows the expression of genes associated with antioxidant activity. (**G**) Pearson correlation analysis between antioxidant indices and BMP/Smads signaling pathway. Bars with no common small letters (a, b, c) indicate statistical differences among the three treatments (*p* < 0.05).

Previous studies reported a strong association between bone-related diseases and oxidative stress [18,23,25]. Next, oxidative stress-related indicators, including SOD, GSH-Px, and GPX-1 were investigated in broiler plasma and TGPs (Figure 5A–C). The results showed that GSH-Px and T-SOD activities in plasma were significantly lower in the TD group than in the CON group on day 7 (*p* = 0.001 and *p* < 0.001, respectively), day 14 (*p* < 0.001, and *p* < 0.001, respectively) and day 21 (*p* = 0.001 and *p* = 0.018, respectively). The plasma MDA level in the TD group was considerably higher than that in the CON group on days 7, 14, and 21 (*p* < 0.001, *p* < 0.001, and *p* < 0.001, respectively). Subsequently, it was found that MOP could reverse the abnormal changes of oxidation indexes caused by TD, and plasma T-SOD and GSH-Px activities were higher in the MOP group than in the TD group on day 14 (*p* = 0.015 and *p* < 0.001, respectively) and day 21 (*p* = 0.229 and *p* = 0.001, respectively), while the content of MDA was significantly decreased compared to that of the TD group on day 14 (*p* = 0.014) and day 21 (*p* < 0.001), returning to the level similar to that of the CON group.

The antioxidant-related genes SOD and GPX-1 in the TGP were further investigated by RT-qPCR. During the whole experiment, SOD and GPX-1 genes in the three groups showed high dispersion along the PC1 and PC2 axes of the PCA score plot (Figure 5D). Moreover, compared with the CON group, the mRNA expression levels of SOD and GPX-1 in the TD group were significantly down-regulated on day 7 (*p* = 0.018 and *p* = 0.021, respectively), day 14 (*p* = 0.014 and *p* = 0.008, respectively) and day 21 (*p* = 0.011 and *p* = 0.003, respectively). After MOP treatment, the mRNA expression levels of SOD and GPX-1 in the MOP group were significantly higher than those in the TD group on day 14 (*p* < 0.001 and *p* = 0.002, respectively) and day 21 (Figure 5E) (*p* = 0.010 and *p* < 0.001, respectively). The heatmap showed more clearly the differences in gene expression levels of SOD and GPX-1 in each group (Figure 5F). Pearson correlation analysis revealed that BMP-2 gene was positively correlated with GPX-1 and MDA (r = 0.443, *p* = 0.030; r = 0.413, *p* = 0.045; respectively), and negatively correlated with T-SOD and GSH-Px (r = −0.417, *p* = 0.043; r = −0.571, *p* = 0.004; respectively). Smad4 was significantly positively correlated with GPX-1 and MDA (r = 0.787, *p* < 0.001; r = 0.474, *p* = 0.019; respectively), and negatively correlated with GSH-Px (r = −0.618, *p* = 0.001; Figure 5G). These results suggest that MOP improves tibial growth in TD broilers in association with oxidative stress.

### 3.5. Effect of MOP on Gut Microbiota of TD Broilers

**Figure 6:**
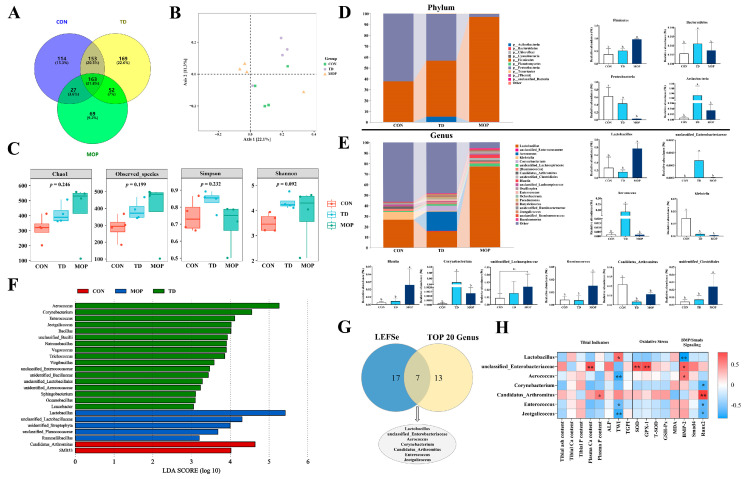
Effects of MOP on the gut microbiota of broilers. (**A**) The Venn diagram analysis of OTUs. (**B**) PCoA is based on the unweighted−UniFrac distance matrix of OTUs. (**C**) Chao1 diversity; observed_species; Simpson; Shannon diversity. (**D**) Relative abundance of gut microbiota composition at the level of phylum. (**E**) Relative abundance of gut microbiota composition at the level of genus. (**F**) LDA scores show the significant bacterial differences in the (log LDA > 3.0; *n* = 4 chickens/group). (**G**). The Venn diagram for LEfSe and the abundance of the top 20 bacterial taxa at the genus level. (**H**) Correlation analysis of tibia-related parameters, bone metabolism-related parameters, oxidative stress and BMP/Smads signaling pathway with gut microbiota. Bars with no common small letters (a, b) indicate statistical differences among the three groups (*p* < 0.05). ns showed no statistical significance among the three treatment groups (*p* > 0.05).

Gut microbiota plays a vital role in bone health, bone aging, and pathological bone loss [21]. We next evaluated whether there was an MOP effect on microbial communities of TD broilers using 16S sequencing. The results indicated that 747 operational taxa (OTUs) were observed in the three experimental groups on day 21, with 114 unique elements in the CON group, 169 unique elements in the TD group, and 69 unique elements in the MOP group (Figure 6A). PCoA analysis (beta diversity) showed significantly different clustering of gut microbiota in the three experimental groups (Figure 6B). In addition, there were no significant differences in microbiome between the experimental groups of broiler chickens in terms of alpha diversity (Figure 6C).

To profile the specific changes in gut microbiota, we analyzed the relative abundance of the predominant taxa (top 10 in phylum and top 20 in genus). At the phylum level, the relative abundance of Firmicutes were significantly increased in the MOP group (*p* < 0.001 and *p* = 0.001, respectively), while the relative abundance of Proteobacteria decreased significantly (*p* < 0.001 and *p* = 0.004. respectively) compared to the CON and TD groups (Figure 6D). The abundance of Actinobacteria were significantly elevated in the TD group than in the CON and MOP groups (*p* = 0.013 and *p* = 0.016. respectively). Bacteroidetes were not significantly different among the three groups. At the genus level, the gut microbiota of the three groups were dominated by *Lactobacillus*, *unclassified_Enterobacteriaceae*, *Aerococcus*, *Klebsiella*, *Corynebacterium*, *unidentified_Lachnospiraceae*, *Ruminococcus*, *Candidatus_Arthromitus*, *unidentified_Clostridiales*, and *Blautia* (Figure 6E). Compared with the CON and MOP groups, the relative abundance of *Aerococcus* (*p* < 0.001 and *p* < 0.001, respectively), *Corynebacterium* (*p* = 0.013 and *p* = 0.014, respectively), and *unclassified_Enterobacteriaceae* (*p* = 0.001 and *p* = 0.001, respectively) were significantly increased; *Candidatus_Arthromitus* (*p* < 0.001 and *p* = 0.035, respectively) were significantly decreased in the TD group. Compared with the CON and TD groups, the *Lactobacillus* (*p* = 0.004 and *p* = 0.001, respectively), *Ruminococcus* (*p* = 0.048 and *p* = 0.042, respectively), *unidentified_Clostridiales* (*p* = 0.024 and *p* = 0.045, respectively), and *Blautia* (*p* = 0.027 and *p* = 0.037, respectively) were significantly increased in the MOP group. Next, linear discriminant analysis effect size (LEfSe) analysis of bacterial community (LDA score > 3.0) at genus level revealed that 24 differential bacterial taxa were identified between the CON, TD, and MOP groups (Figure 6F).

To screen for significant and critical bacterial taxa, we identified seven bacterial taxa including *Lactobacillus*, *unclassifed_Enterobacteriaceae*, *Aerococcus*, *Corynebacterium*, *Candidatus_Arthromitus*, *Enterococcus*, and *Jeotgalicoccus* from the 24 differential bacterial taxa screened by LEfSe analysis and the top 20 bacteria at the genus level by Venn analysis (Figure 6G). In addition, Spearman correlation analysis of the seven screened key bacterial taxa with bone metabolism-related indicators, oxidative stress-related indicators, and BMP/Smads signaling pathway-related genes showed significant positive correlations between plasma Ca content and *unclassified_Enterobacteriaceae* (r = 0.711 and *p* = 0.010), plasma P content and *Candidatus_Arthromitus* (r = 0.580 and *p* = 0.048); tibial weight index showed significant positive correlations with Lactobacillus (r = 0.615 and *p* = 0.033); oxidative stress indicators SOD and GPX-1 genes showed significant positive correlations with *unclassified_Enterobacteriaceae* (r = 0.713 and 0.743, *p* = 0.009 and 0.006, respectively); BMP-2 gene showed significant positive correlations with *unclassified_Enterobacteriaceae* and *Aerococcus*(r = 0.678 and 0.587, *p* = 0.015 and 0.045, respectively), Runx2 gene showed significant positive correlations with *Candidatus_Arthromitus* (r = 0.804 and *p* = 0.002), while BMP-2 gene showed negative correlations with Lactobacillus (r = −0.818 and *p* = 0.001) and Runx2 with Corynebacterium (r = −0.671 and *p* = 0.017), Enterococcus (r = −0.615 and *p* = 0.033) and *Jeotgalicoccus* (r = −0.631 and *p* = 0.028) (Figure 6H). These results indicated that MOP can improve the gut microbiota by increasing the beneficial bacteria count and decreasing the harmful bacteria count, and participating in the regulation of oxidative stress and BMP/Smads signaling pathway in TD broilers.

## 4. Discussion

**Figure 7:**
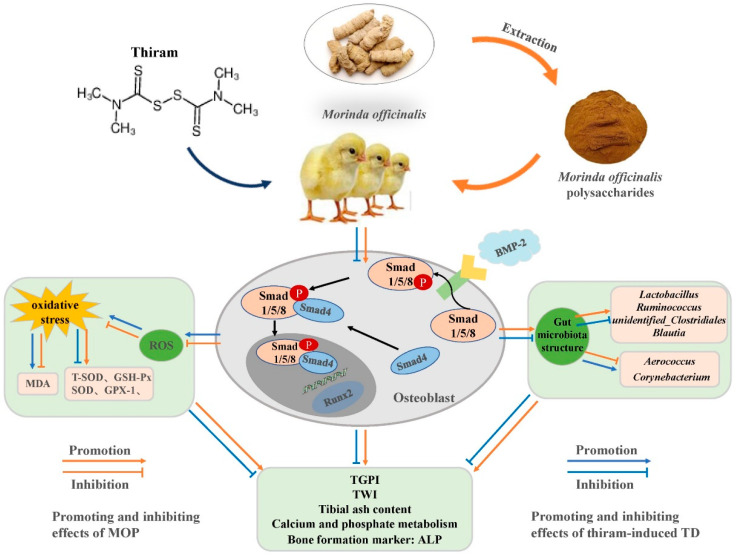
The schematic diagram of the mechanism of MOP in improving TD broilers by regulating the BMP/Smads signaling pathway. ALP, Alkaline phosphatase; BMP-2, Bone morphogenetic protein-2; GSH-Px, Glutathione peroxidase; MDA, Malondialdehyde; Runx2, Runt-related transcription factor-2; ROS, Reactive oxygen species; Smad1,4,5,8, Smad family member 1,4,5,8; TGPI, Tibial growth plate index; T-SOD, Total super-oxide dismutase; TWI, Tibial weight index.

TD is a susceptible disease of the tibial tarsus affecting the proximal growth plate of the tibia in fast-growing poultry, and its prevention and treatment have not been well defined. Numerous studies have confirmed that traditional Chinese herbal medicine plays an important role in improving bone development, such as total flavonoids of *Rhizome drynariae* [37], *Puerarin* [38,39], and *Astragalus membranaceus* [18]. We investigated the therapeutic effects of MOP on TD broilers, and the results showed that MOP could improve tibia-related indicators through BMP/Smads signaling pathway, improve the disturbance of Ca and P metabolism caused by TD, and increase the antioxidant capacity of the chickens. Similarly, MOP could also improve the gut microbiota structure of TD broilers, increase the abundance of beneficial bacteria, inhibit the abundance of harmful bacteria, and achieve the goal of treating TD (Figure 7).

Normal bone development is crucial to broiler growth. Under genetic selection, feeding methods and for other reasons, resulting in broilers growing too fast, this raises the load on the legs of broilers [5,40]. TD is a relatively common leg disease, including changes in weight and length of the tibia, damage to the growth plate of the tibia, and the abnormality of Ca and P metabolism [3]. As two common indices of tibial growth performance, the tibial weight index and tibial growth plate index have been widely used to evaluate the overall skeletal development of broilers. In this study, broilers in the TD group showed significant clinical symptoms, such as depression, leg distortion, lameness, dystasia, inappetency, and the tibial growth plate index was significantly increased. The most apparent response to low growth performance in TD broilers is a clear uncalcified region in the TGP. Blocked metabolism of osteoblasts and osteoclasts and accumulation in the growth plate are the direct causes of clear uncalcified embolism in TD broilers [7]. This is consistent with previous study on growth plate loss in broilers [41]. During the development of tibia, vascular infiltration in the growth plate region is essential for bone growth and calcification. Vascular infiltration can bring the nutrients and hormones necessary for bone development, promote the normal development of chondrocytes and the normal secretion of related proteins, so as to ensure normal calcification. Reduced vascular infiltration leads to disturbed cell differentiation, which affects cartilage calcification and the formation of cartilage thrombus [7,36]. In our study, MOP could promote vascular infiltration in the tibial growth plate region of TD broilers and restore the morphological structure of chondrocytes. Tibial ash content is a common marker of broiler. Ca and P content and the ash content of the tibia directly reflect the texture of bone; the higher the ash content of the tibia, the higher the inorganic mineral content of the bone, or bone density [42,43]. Plasma Ca content, P content is an indicator of Ca and P content in the body, which is closely related to bone growth and bone metabolism. In bone metabolism research, ALP activity is an index reflecting the function and differentiation degree of osteoblasts and can be used as an effective parameter to monitor the changes in bone formation [40,44]. Our results indicated that tibial bone strength, Ca and P metabolism disorder, and bone formation marker ALP decreased in TD broilers. The results of this study are consistent with the findings of Xu et al. [37] (2018) and Liu et al. [42] (2021). Many studies have proved that TCM has the function of tonifying the kidneys and strengthening the bones. For example, TFRD could improve the bone formation and mineralization of tibia in rats [45] and astragalus could enhance bone formation in osteoporosis rats [46]. As a kind of TCM for kidney tonifying and bone strengthening, MOP could promote the proliferation and differentiation of osteogenic cells and inhibit the activity of osteoclasts, and then, indirectly promote bone formation and inhibit bone absorption [47]. In this study, MOP could alleviate the clinical symptoms of TD, balance the disorder of Ca and P metabolism in the body, increase the activity of the plasma ALP, and significantly aid in the growth of the tibia. 

Osteoblast and osteoclast metabolism impairment and accumulation in growth plates are the direct causes of transparent uncalcified embolism in TD broilers [7]. Thus, promoting the proliferation and differentiation of osteoblasts and inhibiting the proliferation and differentiation of osteoclasts are essential elements for treatment with TD. BMP/Smads signaling pathway is the critical signaling pathway in bone formation and has been confirmed to affect osteoblast differentiation. BMP-2BMP-2 plays a vital function in the growth, development, regeneration, and repair of bone by promoting the differentiation of BMSCs into osteoblasts and producing factors related to bone and chondrogenesis [10,11]. In the BMP/Smads signaling pathway, Smad4 plays an important role in coordinating the other Smads molecules. Runx2 is a specific marker for osteogenesis that regulates the proliferation, maturation, and differentiation of osteoblasts, thereby indirectly regulating endochondral and intramembrane osteogenesis [48,49]. In addition, MOP could promote osteoblast proliferation and maturation, inhibit osteoclast differentiation, and regulate bone formation through the BMP-2BMP-2/Runx-2/Osterix pathway [35,47]. MOP was found to restore TD-induced downregulation of BMP/Smads signaling pathway-related genes. This suggests that MOP can improve osteoblast activity by regulating the expression of BMP/Smads signaling genes and thus improve bone injury in TD broilers.

There are many factors causing bone damage, such as oxidative stress, inflammation, apoptosis, etc. Oxidative stress plays a significant role in inhibiting bone growth and development as the most common mechanism in the body. Previous study has shown that oxidative stress plays a core role in the occurrence and development of postmenopausal osteoporosis [50], and osteoarthritis is also correlated with oxidative stress and ROS production [51]. T-SOD and GSH-Px can determine the antioxidant capacity of the body. Several enzyme antioxidant systems, including SOD and GSH-Px, contribute to removing excessive oxygen radicals from the body and reducing oxidative damage. Elevated MDA values, however, indicate that cells are in serious stress and their membranes are becoming more fragile [16,52,53]. We found that T-SOD and GSH-Px levels were decreased, but MDA content was increased, and the expression of SOD and GPX-1 mRNA was decreased in the TD group, indicating that the antioxidant system of TD broilers was damaged. Oxidative stress may be involved in the abnormal bone development process of TD broilers. However, oxidative stress affecting bone growth and development still needs further research. Our results found that MOP can improve the antioxidant capacity of TD broilers, which also confirmed that MOP is a natural antioxidant and free radical scavenger [54,55]. The correlation analysis between oxidative stress and BMP/Smads signaling pathway showed that oxidative stress is associated with BMP/Smads signaling pathway. Some studies have shown that diphlorethohydroxycarmalol may protect MC_3_T_3_-E_1_ osteoblasts from oxidative damage and apoptosis induced by H_2_O_2_ by stimulating the BMP signaling pathway promoting osteoblast differentiation, while up-regulation of LncRNA SNHG15 expression can improve redox balance through the TGFβ/Smad signaling pathway, promote osteogenic differentiation of bone marrow mesenchymal stem cells under oxidative stress, and inhibit their adipogenic differentiation [56,57]. This further demonstrates that oxidative damage can be regulated through the BMP/Smads signaling pathway.

Microbiota in the gut plays an important role in host health [58]. Previous studies have shown that skeletal diseases are associated with gut microbiota disorders, such as osteoarthritis and osteoporosis [19,21]. A few studies have shown that kidney-strengthening and bone-strengthening TCM not only acts on bones, but also improves gut barrier function, regulates gut microecology, optimizes bacterial community structure, and promotes the absorption and metabolism of nutrients to ensure healthy growth of bones [20,59]. In addition, gut microbiota can also release estrogen analogs, serotonin, and other small molecules to affect Ca and P metabolism [33]. Lactobacillus is one of the most critical members of probiotics. It is also the leading group of bacteria in the gut membrane barrier. It has been shown that the *lactobacillus rhamnosus JYLR-005* can prevent the thiram-induced improvement in bone correlation [42]. As one of the body’s beneficial bacteria, *Blautia* provides energy for the body, reduces inflammation, and is associated with host health [60]. Studies have shown that *Ruminococcus* can stabilize the gut barrier and is one of the most effective bacteria in decomposing carbohydrates [61]. *Candidatus_Arthromitus* plays a vital role in the maturation of the host immune system, and its colonization in the gut tract is beneficial to host resistance to foreign pathogens [62]. Our study found that MOP can improve the gut microbiota structure of TD broilers and increase the abundance of *Lactobacillus, Blautia, Ruminococcus,* and *Candidatus_Arthromitus*, thus playing an important role in broiler bones. It is worth noting that the abundance of *Aerococcus* and *Corynebacterium* of broiler chickens in the TD group is significantly higher than that in the other two groups. Several studies have shown that *Aerococcus* and *Corynebacterium* can cause infection of bones and joints, leading to osteomyelitis and suppurative arthritis [63,64,65]. In subsequent correlation analysis, all screened bacteria were found to be associated with the BMP/Smads signaling pathway-related genes, thus we hypothesized that the gut microbiota may be involved in the regulatory process of bone metabolism by affecting the expression of the BMP/Smads signaling pathway. However, the underlying mechanisms still need to be demonstrated by further studies.

## 5. Conclusions

In conclusion, MOP can regulate bone formation through the BMP/Smads signaling pathway, attenuating oxidative stress and regulating the gut microbiota of TD broilers, so as to achieve the effect of treating TD, which provides a valuable reference for further exploration of the treatment of MOP on the prevention and treatment of poultry leg disease, while MOP can be used as a potential target medicine to treat thiram-induced tibial dyschondroplasia. 

## Figures and Tables

**Table 1:** Composition and nutrient level of basal diet.

Ingredients, %	Content
Corn	57.0
Soybean meal	31.5
Corn gluten meal	3.4
Soybean oil	3.1
Limestone	1.2
Dicalcium phosphate	2.0
L-Lysine	0.3
DL-Methionine	0.2
Sodium chloride	0.3
Premix ^1^	1.0
Total	100.0
Calculated nutrient levels	
Apparent metabolizable energy, MJ/kg	12.6
Crude protein, %	21.3
Calcium, %	1.0
Total phosphorus, %	0.7
Available phosphorus, %	0.5
Lysine, %	1.2
Methionine, %	0.5
Methionine + cystine, %	0.9
Analyzed nutrient levels ^2^	
Gross energy, MJ/kg	15.5
Crude protein, %	20.8
Calcium, %	1.1
Total phosphorus, %	0.7
Lysine, %	1.2
Methionine, %	0.5

^1^ Premix provided per kilogram of diet: vitamin A (transretinyl acetate), 10,000 IU; vitamin D3 (cholecalciferol), 3000 IU; vitamin E (all-rac-α-tocopherol), 30 IU; menadione, 1.3 mg; thiamin, 2.2 mg; riboflavin, 8 mg; nicotinamide, 40 mg; choline chloride, 600 mg; calcium pantothenate, 10 mg; pyridoxine·HCl, 4 mg; biotin, 0.04 mg; folic acid, 1 mg; vitamin B_12_ (cobalamin), 0.013 mg; Fe (from ferrous sulfate), 80 mg; Cu (from copper sulphate), 8.0 mg; Mn (from manganese sulphate), 110 mg; Zn (from zinc oxide), 60 mg; I (from calcium iodate), 1.1 mg; Se (from sodium selenite), 0.3 mg. ^2^ Results are the average values of triplicate measurements.

## Data Availability

Data is contained within the article.

## Supplementary Materials

The following supporting information can be downloaded at: https://www.mdpi.com/article/10.3390/metabo12100958/s1, Table S1: Primers used for the RT-qPCR.
