# Supplementary material for: Morinda officinalis Polysaccharides Ameliorates Bone Growth by Attenuating Oxidative Stress and Regulating the Gut Microbiota in Thiram-Induced Tibial Dyschondroplasia Chickens
Source: Metabolites. 2022 Oct 10;12(10):958. doi: 10.3390/metabo12100958 (PMC9609565; doi:10.3390/metabo12100958)
Supplement: Supplementary file 1 [file metabolites-12-00958-s001.zip › metabolites-1920891-supplementary.pdf]

Morinda officinalis polysaccharides ameliorate bone growth by attenuating oxidative stress and regulating the gut microbiota in thiram-induced tibial dyschondroplasia chickens

Table S1. Primers used for the RT-qPCR

Genes	Accession number	Primer sequence (50-30)	Product size（bp）
GAPDH	NM_204305.1	F: GCCCAGAACATCATCCCA	137
		R: CGGCAGGTCAGGTCAACA	
BMP-2	XM_015283435.1	F: TCAGCTCAGGCCGTTGTTAG	185
		R: ACCCCACGTCATTGAAGTCC	
Runx2	AF_445419	F: TAAAGGTGACGGTGGATGG	190
		R: TGTGGATTAAAAGGACTTGGTG	
Smad4	XM_040690690.1	F: CGACCTGCACAAAAACGAACTGAA	107
		R: GCAGACGCTGTCGTATTTGAG	
SOD	NM_205064.1	F: CCGGCTTGTCTGATGGAGAT	125
		R: TGCATCTTTTGGTCCACCGT	
GPX-1	NM_001277853.2	F: ACGGCGCATCTTCCAAAG	73
		R: TGTTCCCCCAACCATTTCTC	

Note: F. Forward primer; R. Reverse primer.;
